# Physical exercise and mental health among older adults: the mediating role of social competence

**DOI:** 10.3389/fpubh.2024.1385166

**Published:** 2024-06-19

**Authors:** Bin Hou, Yuxin Wu, Yuqi Huang

**Affiliations:** ^1^School of Cultural Tourism and Public Administration, Fujian Normal University, Fuzhou, China; ^2^School of Management, Yang-En University, Quanzhou, China

**Keywords:** physical exercise, mental health, social competence, older adults, mediating role

## Abstract

**Background:**

Healthy aging is crucial to the quality of life of older adults, of which mental health is an essential part. Physical exercise strongly affects their mental health and can alleviate psychological problems to a certain extent. Nevertheless, the correlation between physical exercise and the mental health of older adults individuals, as well as the underlying mechanism by which physical exercise impacts mental health, remains rather ambiguous.

**Methods:**

We utilized multiple linear regression models to investigate the relationship between physical activity and mental health in 3,240 persons aged 60 and up. The propensity score matching (PSM) method was used to assess the robustness of the regression results. In addition, sequential recursive models were employed to investigate the mediating role of social competence in the link between physical activity and mental health.

**Results and discussion:**

We discovered a strong favorable association between physical exercise and mental health, which was mediated by social competence. Furthermore, the effect of physical exercise on mental health differed across older persons from various socioeconomic backgrounds.

**Conclusion:**

Older adults should strengthen their understanding of the role of physical exercise. Sports organizations that serve older adults by providing resources and services to help them maintain physical fitness and by hosting sports activities and competitions according to their needs should be established.

## Introduction

Population aging will increase in the future decades (1). China has a large older adult population, and its population is aging faster than that in any other country (2). These demographic trends have promoted medical research on the health problems of older adults. Researchers have studied the relationship between age structure and self-assessed health and the health trajectory of older adult population (3) as well as the relationship between aging and medical care plans (4).

As older adults age, their physical functions gradually deteriorate, which often negatively affects their psychological health. Older adults are often unable to adapt to new social roles, living environments, and lifestyle changes, which may result in anxiety, depression, loneliness, and other negative emotions or adaptive psychological disorders (5–7).

Physical exercise strongly affects people’s mental health and can alleviate psychological problems to a certain extent. For example, in a cross-sectional study of individuals aged ≥18 years, researchers discovered that physical activity was associated with lower self-reported mental health burden, and compared with inactivity, all types of physical activity were associated with lower mental health burden (8). In another study, researchers followed up 129 people for 1 year and discovered that exercising with others significantly improved the mental health of middle-aged and older adults (9). Many earlier studies have reported the effects of physical exercise on the psychology of individuals, but their samples were limited to adolescents and adults in specific locations (10–12).

Most of the aforementioned studies were experimental studies, in which the researchers’ statistical analyses failed to account for the problem of endogeneity, weakening the ability of their evidence to support their conclusions. Therefore, in the present study, we investigated the effect of physical exercise on the mental health of older adults and analyzed the underlying mechanisms of the effect from the perspective of social competence.

This study contributes to the relevant literature in the following three aspects. First, we investigated effect of physical exercise on the mental health of older adults, whereas previous studies have focused excessively on teenagers and other groups. Second, our analysis of the mechanism of the effect of physical exercise on the mental health from the perspective of social competence not only further elucidates the relationship between physical exercise and mental health but also may serve as a reference for health management departments developing health interventions. Social competence is an indicator of social engagement, including the frequency and scope of social engagement (13). Third, this study uses data from a sample survey of Chinese society and uses causal inference to overcome the endogeneity problem and sample selection bias. Therefore, the conclusions of this study are more accurate than those of previous studies, which is of great importance for improving the scientific basis and effectiveness of policy design.

## Literature review and hypotheses

Health is a crucial part of human capital. Many health-related studies are based on the health production function framework proposed by Grossman (14, 15), which systematically summarizes the factors affecting health. The framework assumes that an individual’s health level will decrease with age but that health benefits can be obtained through investment, and the factors that determine the scale of health investment include individuals’ lifestyle behaviors, such as physical exercise (16).

Physical exercise is a common health investment behavior with obvious mental health benefits (11, 17). A study examined the correlation between regular physical activity and depressive symptoms in 256 Korean individuals aged 65 years or older who participated in the 2014 Korea National Health and Nutrition Examination Survey. The researchers found that consistent flexibility in physical activity assists older adults in maintaining a healthy mental state (18). The researchers of another study reported that an 8-week adapted physical activity intervention program improved the mental health of 30 older adult women who were randomly assigned to a control group or a training group and found dynamic lifestyle including regular physical activity was key to maintaining the psychological health of older adults as they age (19).

Drawing on previous research findings, we predicted that physical exercise may exert a positive effect on the mental health of older adults. Accordingly, we developed the following hypotheses:

*H1*: Physical exercise exerts a positive effect on the mental health of older adults.

We further considered the mechanism by which physical exercise can promote the mental health of older adults. The aforementioned studies have directly investigated the effect of physical exercise on individual mental health, but they have not conducted theoretical analysis or empirical investigations of the mechanisms underlying this effect. According to the theory of perceived social isolation, if an individual perceives that they are socially isolated, their overall cognitive performance worsens, and they will experience negative emotions and depressive episodes more frequently, and their sensitivity to social threats will increase (20). Some empirical studies have supported these theoretical views (21, 22).

Social communication is essential to human survival. Active social participation and interpersonal interaction can improve an individual’s quality of life (23, 24). Although the subjective purpose of physical exercise is health investment, physical activity objectively creates opportunities for social interaction, and people who participate in such activities often engage in extensive and in-depth interactions in the process. Some studies have also demonstrated a link between physical activity and social skills. Taking social residents aged ≥50 years as the research object, researchers discovered that people who engage in physical exercise for longer time are more likely to be happy. Participating in sports can expand an individual’s social network, thus promoting their accumulation of social capital and their social skills (25). In addition, in a study of Chicago residents, researchers discovered that community physical activity helped strengthen neighborhood bonds and improved people’s social skills (26). Therefore, in this study, we examined the mechanism underlying the effect of physical exercise on the mental health of older adults from the perspective of social competence.

In a 2008 study of older adults from the general population in Finland in 2008, researchers discovered that social support and social interaction are important factors that should be considered when designing interventions to promote mental health and prevent mental disorders among older adults (27). A study of 947 older adults people in a Swiss community found that social engagement, including social competence, can intervene in their depressive symptoms and improve their mental health (28). Therefore, we speculated that social competence is a key mechanism through which physical exercise promotes the mental health of older adults; thus, we developed the following hypothesis:

*H2*: Physical exercise promotes the mental health of older adults by encouraging social competence.

## Methods

### Data and study sample

The data utilized in this study was obtained from the 2018 China General Social Survey (CGSS), administered by the China Survey and Data Center of Renmin University of China. In the survey, the total sample size of CGSS 2018 database is 12,787. The purpose of the survey is to comprehensively and systematically describe and analyze Chinese society through the annual survey data, and reveal the social changes in China and the changes in the roles and concepts of social members. Stratified sampling was applied to collect data on the health status, lifestyle, social attitudes, and work status of Chinese residents in 28 provinces (municipalities and autonomous regions) of China. The research subjects of the present study were the older adult population. To identify older adults, we calculated their age by subtracting their birth year from 2018. We then selected individuals whose age was equal to or more than 60 years. After removing samples with missing values for the independent, dependent, and control variables, we got a total of 3,240 valid samples.

### Measures

#### Dependent variable

The study focused on the mental well-being of older adults individuals as the dependent variable. The question “How often have you felt depressed in the past 4 weeks?” was used to measure the dependent variable. Responses were provided in a 5-point scale ranging from “always” to “never.” Higher values indicated better mental health. This is consistent with the study variables and conceptual operationalization used by the other researcher (29).

#### Independent variable

The independent variable of this study was physical exercise. The question “In the past year, how often have you participated in physical exercise in your free time?” was used to measure the independent variable. The available choices were as follows: 1 = daily, 2 = multiple times per week, 3 = multiple times per month, 4 = multiple times per year, and 5 = never. We reverse-coded the options 1–5 so that the higher the value, the higher the frequency of participating in physical exercise.

#### Mediating variable

The mediating variable of this study was social competence. The following questions were used to measure the mediating variable: “How often have you spent your free time with relatives who do not live together in the past year?” for which the options were reverse-coded as 0 = never, 1 = multiple times per year or less, 2 = multiple times per month, 3 = multiple times per week, and 4 = daily; “How often have you gotten together with friends in your free time in the past year?” for which the options were reverse-coded as 0 = never, 1 = multiple times per year or less, 2 = multiple times per month, 3 = multiple times per week, and 4 = daily; “In the past year, how often have you socialized/visited others in your free time?” for which the options were reverse-coded as 0 = never, 1 = rarely, 2 = sometimes, 3 = often, and 4 = very often; “How often do you engage in social entertainment activities with your neighbors?” for which the options were reverse-coded as 0 = never, 1 = less than once a year, 2 = multiple times a year, 3 = roughly once a month, 4 = multiple times a month, 5 = once or twice a week, and 6 = nearly every day; and “How often do you engage in social entertainment activities with other friends?” for which the options were reverse-coded as 0 = never, 1 = less than once a year, 2 = multiple times a year, 3 = roughly once a month, 4 = multiple times a month, 5 = once or twice a week, and 6 = nearly every day. We summed each participant’s scores for these five questions together to obtain their overall social competence score. Higher scores indicated stronger social competence.

#### Control variables

We selected control variables, including individual, family, variables and social characteristic variables, from the characteristics of older adults assessed on the CGSS. The individual characteristic variables recorded were age, sex (0 = female, 1 = male), education level (0 = illiterate, 1 = elementary school, 2 = junior, middle, and high school, 3 = university or higher), and employment status (0 = not employed, 1 = employed). The family characteristic variables were number of children, whether an individual lived with their partner (0 = living separately, 1 = living together), and living area. The social characteristic variables were residence (0 = rural, 1 = urban), Internet usage (0 = does not use the Internet, 1 = uses the Internet), personal socioeconomic status (1 = low, 2 = middle, 3 = upper), endowment insurance (0 = insured, 1 = not insured), and health insurance (0 = insured, 1 = insured).

### Statistical analyses

Stata 16.0 was used to investigate the impact of physical exercise on the mental health of older persons. First, we used descriptive statistics to assess the distributions of the dependent, independent, intermediate, and control variables. Second, we analyzed the association between physical exercise and mental health using a multiple linear regression model, followed by an ordered probit model and propensity score matching to ensure the regression results were robust. Third, we used sequential mediation analysis to assess the mediating role of social competence. Finally, we investigated the variability of the effect of physical exercise on the mental health of older persons from various socioeconomic backgrounds.

## Results

### Descriptive analysis

Tables 1, 2 presents the characteristics of the CGSS 2018 sample used in this study (*N* = 3,240), including the value range, mean and standard deviation for each continuous variable and the variable definitions, frequency and percentage for each categorical variable. The mean mental health score was 3.875, indicating that the mental health status of most of the respondents was at the medium level. 53.12% of the respondents engaged in regular physical exercise. In addition, the mean number of children was 2.226, and the mean living area was approximately 100.617 m^2^.41.09% of the respondents had junior, middle and high school, 53.4% of the respondents were male, 99.35% were married, 26.88% were employed, and 91.7% lived with their partners. A total of 33.36% of the respondents reported using the Internet, and approximately 50% of the respondents had rural and urban household registrations. Most of the respondents had a middle or low socioeconomic status; 85.15% had endowment insurance, and 93.95% had health insurance.

**Table 1:** The characteristics of continuous variables.

Continuous variables	Value range	Mean	SD
Mental health	1–5	3.875	0.981
Social competence	0–24	9.742	5.300
Age	60–118	68.138	6.573
Number of children	0–14	2.226	1.266
Living area	7–300	100.617	56.584

**Table 2:** The characteristics of categorical variables.

Categorical variables	Variable definitions	Frequency	Percent
Physical exercise	0 = no	1,519	46.88%
	1 = yes	1,721	53.12%
Sex	0 = female	1,510	46.60%
	1 = male	1,730	53.40%
Marry	0 = unmarried	21	0.65%
	1 = married	3,219	99.35%
Education level	0 = illiterate	746	23.02%
	1 = elementary school	923	28.49%
	2 = junior, middle and high school	1,330	41.05%
	3 = university or higher	240	7.44%
Employment status	0 = no	2,369	73.12%
	1 = yes	871	26.88%
Living situation	0 = living separately	269	8.30%
	1 = living together	2,971	91.70%
Internet usage	0 = no	2,159	66.64%
	1 = yes	1,081	33.36%
Residence	0 = rural	1,634	50.43%
	1 = urban	1,606	49.57%
Socioeconomic status	1 = low	1,776	54.81%
	2 = middle	1,248	38.52%
	3 = upper	216	6.67%
Endowment insurance	0 = no	481	14.85%
	1 = yes	2,759	85.15%
Health insurance	0 = no	196	6.05%
	1 = yes	3,044	93.05%

### Effect of physical exercise on mental health

In this study, we conducted stepwise regression to analyze the effect of physical exercise on the mental health of older adults. Table 3 presents the specific regression results. Model 1 is the regression model without any control variables. Model 2 includes individual characteristic variables, Model 3 includes individual and family characteristic variables, and Model 4 includes individual, family, and social characteristic variables. The regression coefficients for physical exercise in Models 1, 2, 3, and 4 were 0.442, 0.317, 0.293, and 0.166, respectively, indicating that physical exercise exerts a significant and positive effect on the mental health of older adults at the 1% significance level.

**Table 3:** The effect of physical exercise on the mental health of older adults.

	Model 1	Model 2	Model 3	Model 4
	Mental health	Mental health	Mental health	Mental health
Physical exercise	0.442***	0.317***	0.293***	0.166***
	(0.034)	(0.036)	(0.036)	(0.037)
Age		0.523	0.534	0.56**
		(0.512)	(0.451)	(0.277)
Sex (female)				
Men		0.099***	0.097***	0.117***
		(0.035)	(0.035)	(0.035)
Education level (illiterate)				
Elementary school		0.143***	0.137***	0.097**
		(0.048)	(0.048)	(0.047)
Junior, middle and high school		0.367***	0.341***	0.211***
		(0.047)	(0.048)	(0.051)
University or higher		0.534***	0.489***	0.246***
		(0.074)	(0.076)	(0.082)
Employment status (no)				
Yes		−0.049	−0.036	0.018
		(0.040)	(0.041)	(0.042)
Number of children			−0.052***	−0.036**
			(0.015)	(0.016)
Living situation (living separately)				
Living together			0.010	0.003
			(0.063)	(0.062)
Living area			0.000	0.000
			(0.000)	(0.000)
Internet usage (no)				
Yes				0.150***
				(0.042)
Residence (rural)				
Urban				0.147***
				(0.045)
Socioeconomic status (Low)				
Middle l				0.302***
				(0.035)
Upper				0.303***
				(0.069)
Endowment insurance (no)				
Yes				−0.068
				(0.050)
Health insurance (no)				
Yes				−0.040
				(0.073)
Constant term	3.703***	3.027***	3.040***	3.045***
	(0.025)	(0.229)	(0.232)	(0.238)
*N*	3,240	3,240	3,240	3,240
*R* ^2^	0.027	0.064	0.067	0.099

*p < 0.10, **p < 0.05, ***p < 0.01; Values in brackets are standard errors.

Furthermore, the Model 4 regression findings showed that age had a favorable effect on the respondents’ mental health at the 5% significant level. This could be because when people get older, they experience less stress. At the 1% significance level, male respondents reported better mental health than female respondents. The respondents’ mental health was also significantly and positively affected by their level of education. At the 5% level, there was a strong negative association between the number of children and the older adult’s mental health. Internet use had a substantial and beneficial effect on respondents’ mental health at the 1% significance level. The average mental health score of respondents who resided in cities was greater than that of participants who lived in rural areas. Individual socioeconomic position was found to be strongly and positively linked with respondents’ mental health at the 1% level.

### Robustness test

#### Ordered probit model test

The dependent variable in this study was mental health, which was rated on a scale of 1–5 and treated as a discrete and ordered variable. To perform a robustness test on the regression results, we built an ordered probit model (Table 3). The robustness test results (Table 4) were consistent with the regression results (Table 3); the effects of physical exercise on mental health in Models 1–4 in Tables 3, 4 were positively associated at the 1% significance level, suggesting the regression results’ robustness.

**Table 4:** Robustness test results.

Variables	Model 1	Model 2	Model 3	Model 4
Physical exercise	0.359*** (0.038)	0.230*** (0.040)	0.213*** (0.041)	0.126*** (0.042)
Control variables	No control	Individual variables	Individual and family variables	All control variables
*N*	3,240	3,240	3,240	3,240
LR statistic	89.83***	218.15***	229.17***	340.88***
Pseudo *R*^2^	0.010	0.025	0.027	0.040

*p < 0.10, **p < 0.05, ***p < 0.01; Values in brackets are standard errors.

#### Propensity score matching

Our findings were influenced by sample selection bias due to confounding factors that affect both physical exercise and mental health in older persons. Therefore, we employed propensity score matching to rectify the regression results. First, because the independent variable used for propensity score matching was a dummy variable, the samples were divided into two groups: those who reported engaging in physical exercise and those who did not. Second, we matched the samples using the following approach. (1) We condensed the control variables into an index to calculate the likelihood of each sample belonging to the experimental group; (2) We used five matching methods (1-to-1 nearest neighbor, 1-to-4 nearest neighbor, radius, kernel, and local linear regression matching) to match the experimental and control groups, and classified individuals with similar tendency values into the same groups to ensure that members of the same group had similar characteristics; (3) We estimated the average treatment effect (ATT) on the participants to represent the difference in mental health between the experimental and control groups.

Balance test is helpful to judge whether propensity value matching can effectively remove potential confounding factors, so as to improve the accuracy and reliability of research results. If the control group and the experimental group have a good balance of confounding factors, then the comparison results between the two groups can more accurately reflect the effects of the intervention factors, and thus better support the conclusions of the study. Table 5 lists the changes of the main characteristic variables of the older adults population before and after matching. It can be seen that before matching, there are obvious differences in the characteristic variables between the treatment group and the control group, resulting in the inaccurate estimation of the differences in mental health between the two groups of samples. However, the standard deviation after matching is within 10, and most variables are not significant after matching. This shows that the differences between the samples have been greatly eliminated after matching, and the matching effect is good, and the propensity score matching can be carried out.

**Table 5:** Comparison of sample characteristics before and after matching.

Variable	Sample	Mean value		Standardized bias (%)	Bias reduction (%)	*T* value	*p* value
		Treated	Control				
Age	Unmatched	90.442	90.859	−2.7		−0.70	0.506
	Matched	90.416	89.289	1.7	26.3	0.47	0.464
Sex	Unmatched	0.358	0.186	28.2		−1.80	0.073
	Matched	0.260	0.228	3.1	83.7	6.17	0.224
Educational level	Unmatched	0.854	0.703	37.1		11.26	0.000
	Matched	0.853	0.850	0.6	98.5	0.27	0.786
Employment status	Unmatched	0.909	0.951	−16.8		−4.42	0.000
	Matched	0.909	0.853	4.1	−30.2	0.18	0.135
Number of children	Unmatched	4.980	5.329	−21.5		−5.78	0.000
	Matched	4.981	4.636	3.9	45.8	0.53	0.712
Living situation	Unmatched	0.045	0.031	7.2		1.94	0.053
	Matched	0.045	0.026	4.2	−39.4	4.29	0.000
Living area	Unmatched	0.064	0.033	14.4		3.75	0.000
	Matched	0.064	0.065	−0.3	98.1	−0.10	0.922
Internet usage	Unmatched	1.903	1.152	49.1		12.89	0.000
	Matched	1.850	2.230	−2.1	40.6	−0.26	0.309
Residence	Unmatched	9.194	8.204	12.9		3.58	0.000
	Matched	9.136	9.405	−3.5	72.8	−1.32	0.186
Socioeconomic status	Unmatched	5.772	4.906	38.1		10.36	0.000
	Matched	5.772	4.444	3.5	−53.3	0.45	0.576
Endowment insurance	Unmatched	0.151	0.089	19.1		5.08	0.000
	Matched	0.148	0.133	4.7	75.6	1.80	0.171
Health insurance	Unmatched	10.944	11.199	−36.4		−10.12	0.000
	Matched	10.953	10.876	11.0	69.7	4.56	0.000

Table 6 shows the ATT generated using various matching algorithms. The ATT values were more than zero in all models and statistically significant, showing that the findings that physical activity has a considerable favorable impact on the mental health of the older adults are robust.

**Table 6:** Propensity score matching results (ATT).

Dependent variable: Mental health				
		ATT	SE	*t*
Nearest neighbor matching	1 to 1 Matching	3.08***	0.41	4.51
	1 to 4 Matching	3.11***	0.38	5.23
	Radius Matching	3.37***	0.32	5.19
The whole matching	Kernel Matching	3.29***	0.35	4.59
	Local Linear Regression Matching	3.16***	0.37	4.33

***p < 0.01 (*t* > 2.76), **p < 0.05 (*t* > 1.96), *p < 0.1 (*t* > 1.65).

#### Test of the mediating effect of social competence

We used sequential recursive model to analyze the effect of the mediating variable (30). Table 7 presents the mediating effect of social competence. At the 1% level of significance, physical exercise improved the mental health of older individuals in Model 1. Physical exercise had a positive impact on the social competence of older individuals in Model 2, with a significance level of 1%. Model 3 found that social competence is one of the mechanisms by which physical exercise influences the mental health of older adults. At the 1% significance level, both physical exercise and social competence had a significant and positive effect on the mental health of older adults. Specifically, the effect of physical exercise on mental health decreased from 11.6% in Model 1 to 10.5% in Model 3. Thus, the effect of physical exercise on the mental health of older adults is mediated by social competence, and increased physical activity can enhance the mental health of older adults through the improvement of their social skills.

**Table 7:** Physical exercise and mental health of older adults: the mediating role of social competence.

Variables	Model 1	Model 2	Model 3
	Mental health	Social competence	Mental health
Physical exercise	0.116*** (0.037)	1.131*** (0.201)	0.105*** (0.037)
Social competence			0.010*** (0.003)
Constant term	3.045*** (0.238)	8.822*** (1.312)	2.959*** (0.240)
Control variables	All control variables	All control variables	All control variables
*N*	3,240	3,240	3,240
*R* ^2^	0.099	0.065	0.102

*p < 0.10, **p < 0.05, ***p < 0.01; Values in brackets are standard errors.

We tested the mediating effect of social competence by using the Karlson–Holm–Breen (KHB) method (31). The findings are displayed in Table 8. The results presented in Table 7 demonstrate that the direct and total effects of physical exercise on the mental health of older individuals were both statistically significant at the 1% level (mean = 0.324 and 0.311, respectively). Significant at the 1% level, the indirect effect of physical exercise on the mental health of older adults via social competence was 0.013, suggesting that social competence mediates the relationship between physical exercise and older adults’ mental health.

**Table 8:** Mediation test results.

Mediating variables	Effect of type	Coefficient	Standard error	*Z*	*p*
Physical exercise	Total effect	0.324***	0.034	9.53	0.000
	Direct effect	0.311***	0.034	9.09	0.000
	Indirect effect	0.013***	0.004	2.86	0.004

### Heterogeneity analysis

In this section, this study first tested whether the interaction term between physical exercise and personal socioeconomic status significantly affected older adults’ mental health; then, the samples were grouped according to the individual socioeconomic status and divided into three sub-samples of lower-level, middle-level, and upper-level to investigate the impact of physical exercise on mental health. The regression results from Table 9 show that the regression coefficient of the interaction term between physical exercise and personal socioeconomic status is 0.117, which is significant at the 1% level.

**Table 9:** Mediating effect test results.

	Mental health	
Variables	Model 1	Model 2
Physical exercise	0.139****	0.187*
	(0.053)	(0.112)
Personal socioeconomic status	0.134***	0.165*
	(0.051)	(0.098)
Interactive items		0.117**
		(0.054)
Control variables	All control variables	All control variables
*N*	3,240	3,240
R2	0.363	0.356

*p < 0.10, **p < 0.05, ***p < 0.01; Values in brackets are standard errors.

The frequency with which a person engages in physical exercise is affected by their socioeconomic status (32, 33), and mental health is also closely related to their socioeconomic status (34, 35). We compared the effects of physical exercise on the mental health of older adults with high, middle, and low socioeconomic statuses (Table 10). The results indicated that physical exercise exerted a significant effect on the mental health of older adults with a high socioeconomic status at the 1% significance level, and that older adults with a high socioeconomic status could gain more mental health benefits from physical exercise than could older adults with a middle or low socioeconomic status.

**Table 10:** Heterogeneity analysis.

	Lower-level older adults	Middle-level older adults	Upper-level older adults
	OLS	OLS	OLS
Physical exercise	0.160*** (0.051)	0.032 (0.056)	0.258* (0.143)
Control variables	All control variables	All control variables	All control variables
*N*	1,776	1,248	216
*R* ^2^	0.074	0.052	0.175

*p < 0.10, **p < 0.05, ***p < 0.01; Value in brackets are standard errors.

## Discussion

### There is a strong correlation between physical exercise and mental health of older adults

Utilizing 2018 CGSS data, we examined the correlation between physical activity and mental health among older individuals in China in this study. H1 is supported by the fact that we discovered a significant positive correlation between physical exercise and mental health among older adults in China. Specifically, we found that those older adults who engage in physical activity have superior mental health to those who do not. Consistent with previous research findings regarding the correlation between physical activity and mental health, this conclusion is reached. For example, researchers explored the effects of different sports and physical exercise on the mental health of older adults in remote areas of Australia and discovered that physical exercise interventions had significant effects on psychological distress (36). In another study, researchers assessed the effects of physical activity on the mental health of 875 older adults from a city in southern Brazil. They discovered that the older adults who were sedentary were more likely to have depression than were those who were physically active, and that physical activity could reduce the likelihood of or delay depression and dementia (37).

### Social competence plays a mediating role in the relationship between physical exercise and mental health

Social competence mediates the relationship between physical exercise and mental health in a significant way, according to the findings of the present study; that is, social competence is a key mechanism by which physical exercise affects the mental health of older adults, and physical exercise can improve the mental health of older adults by enhancing their social competence. As a result, H2 receives support. This result is consistent with findings from prior research. For example, researchers tracked a group of 41,995 adults from UK population and determined that exercise, smoking, in-person and online social interaction are significant mediators of the effect of mental health on future physical health. In-person social interaction is the largest of these, accounting for 2.3% of the total effect (38). In another study, researchers analyzed the effects of dance activities on the mental health older adults aged 65–84 years and discovered that dance activities helped the participants maintain not only their cognitive, motor, and perceptual abilities but also their physical and mental health by improving their social competence (39). Improved social skills can positively affect the mental health of older adults and, in turn, their physical exercise habits, thus helping them adapt to aging-related challenges.

### Effect of physical exercise on mental health varies among older adults with different socioeconomic statuses

Physical activity has no significant effect on the mental health of older adults with a moderate or low socioeconomic status, according to the findings of the present study. However, this effect is statistically significant for older adults with a high socioeconomic status. As a result of having greater access to exercise opportunities, individuals with higher socioeconomic status engage in physical activity more frequently than those with lower socioeconomic status. This is due to the fact that those with a higher socioeconomic status face fewer challenges in their daily lives and possess greater self-assurance regarding their capacity to manage the rigors of an exercise regimen (33). A low socioeconomic status may also be a risk factor for poor mental health (40). Consequently, additional research is warranted to examine the correlation between socioeconomic status indicators and the fulfillment of personal psychological requirements and mental health (34). The physical exercise– and mental health–related needs of older adults with a middle or low socioeconomic status should be prioritized.

## Limitations

There are limitations to the investigation. Firstly, a valid inference regarding the causal relationship between physical exercise and mental health among senior adults is not possible due to the utilization of cross-sectional survey data from 2018. Secondly, due to the unmeasured personality traits of older individuals, additional confounding variables might have been disregarded. The mechanism of action between physical exercise and mental health in older adults may also be influenced by other hierarchical variables.

## Implications

The results of this study indicate that physical exercise is significantly and positively correlated with the mental health of older adults, and that social competence plays a mediating role in the relationship between physical exercise and mental health among older adults. Accordingly, we propose the following suggestions. First, at the individual level, older adults should strengthen their understanding of the role of physical exercise and should change their attitudes toward physical exercise. Physical exercise helps individuals develop strong social skills and mental health, thereby enhancing their sense of self-efficacy and self-worth. Second, at the social level, sports organizations that serve older adults by providing resources and services to help them maintain physical fitness and by hosting sports activities and competitions according to their needs should be established. Finally, at the national level, the government should implement policies that support national fitness and are designed considering the role of sports in social development, create a positive cultural atmosphere for physical exercise, and encourage people to voluntarily participate in physical exercise. The future research directions are as follows: First, on the basis of exploring the measurement of the concept of social competence in this study, a more comprehensive and standardized index system is formed for further measurement; Second, in terms of the specific mechanism of the impact of physical exercise on the mental health of the older adults, in addition to social competence, whether there are other important influencing factors can be explored.

## Data availability statement

The original contributions presented in the study are included in the article/supplementary material, further inquiries can be directed to the corresponding author.

## Author contributions

BH: Conceptualization, Methodology, Supervision, Writing – original draft. YW: Software, Writing – review & editing. YH: Validation, Writing – review & editing.
